# Efficacy of CalliSpheres® microspheres versus conventional transarterial chemoembolization in the treatment of refractory colorectal cancer liver metastasis

**DOI:** 10.1186/s12885-023-11350-y

**Published:** 2023-10-12

**Authors:** Haitao Li, Xiaolin Zhang, Wenjiang Zhao, Fei Cai, Jia Qin, Jie Tian

**Affiliations:** Department of Interventional Radiology, Yichang Central People’s Hospital, First College of Clinical Medical Science, China Three Gorges University, 183 Yiling Avenue, Yichang, 443003 China

**Keywords:** Refractory colorectal cancer liver metastases, Conventional TACE, CalliSpheres® DEB-TACE, Treatment response, Survival profile

## Abstract

**Objective:**

CalliSpheres® is a microsphere that is already widely used for primary liver cancer treatment; however, its application in colorectal cancer liver metastasis (CRLM) is limited. The current study aimed to investigate the efficacy of CalliSpheres® drug-eluting bead (DEB) transarterial chemoembolization (TACE) therapy versus (vs.) conventional cTACE therapy in treating refractory CRLM (RCRLM) patients.

**Methods:**

Twenty-two RCRLM patients who underwent CalliSpheres® DEB-TACE therapy (*n* = 11) or cTACE therapy (*n* = 11) were retrospectively analyzed. Data on clinical response, progression-free survival (PFS) and overall survival (OS) were retrieved.

**Results:**

The objective response rate (36.4% vs. 18.2%, *P* = 0.338) and disease control rate (81.8% vs. 54.4%, *P* = 0.170) were both numerically (but not statistically) higher in the DEB-TACE group than in the cTACE group. Meanwhile, PFS was prolonged in the DEB-TACE group compared with the cTACE group [median: 12.0 (95% CI: 5.6–18.4) vs. 4.0 (95% CI: 0.9–7.1) months, *P* = 0.018]; OS was also longer in the DEB-TACE group compared with the cTACE group [median: 24.0 (95% CI: 18.3–29.7) vs. 14.0 (95% CI: 7.1–20.9) months, *P* = 0.040]. In addition, after adjustment by multivariate Cox analyses, DEB-TACE was superior to cTACE independently regarding PFS (HR: 0.110, 95% CI: 0.026–0.463, *P* = 0.003) and OS (HR: 0.126, 95% CI: 0.028–0.559,* P* = 0.006).

**Conclusion:**

CalliSpheres® DEB-TACE therapy may prolong survival profile than cTACE therapy in RCRLM patients, while further validation is still needed.

## Introduction

Colorectal cancer ranks as the third most common cancer and second leading cause of cancer-related death globally [1], with an unneglectable proportion of cases are diagnosed with distant metastasis [2]. Liver is the highly prevalent site of colorectal cancer metastases due to the venous drainage from gastrointestinal system that accounts for approximately half of the cases, leading colorectal cancer liver metastasis (CRLM) to be a clinical challenge [3, 4]. The optimal choice for CRLM is still surgical resection; however, most patients lose the chance and systemic as well as locoregional therapies are necessary [5, 6].

Transarterial chemoembolization (TACE) is a locoregional treatment widely used for hepatocellular carcinoma (HCC) [7] and is also applied in CRLM patients [8]. Conventional TACE (cTACE) is performed by injecting chemotherapeutic drugs with carriers (such as lipiodol) and embolized agents into the tumor feeding artery, which is commonly used in clinical practice [9]. Then, along with the development of material science and biotechnology, a new kind of TACE was proposed named drug-eluting bead TACE (DEB-TACE), which uses microspheres to load/release chemotherapy drugs and embolize the tumor feeding artery [10]. DEB-TACE presents several advantages over cTACE, such as permitting fixed drug doses, sustained release, and less leakage into the circulatory system [11, 12], resulting in its superior efficacy and safety compared to cTACE in the treatment of HCC as well as CRLM [13–15].

CalliSpheres® (Jiangsu Hengrui Medicine Co. Ltd., China) is the first microsphere independently developed in China, which is made of polyvinyl ethanol (PVA) and exhibits good loadability, release profile, biocompatibility, and embolizing effects [16, 17]. A recent meta-analysis comprehensively revealed that CalliSpheres® DEB-TACE therapy realized higher rates of complete response (CR), objective response rate (ORR), and disease control rate (DCR) and achieved a trend of longer survival compared to cTACE therapy in HCC patients [18]. In addition, CalliSpheres® DEB-TACE therapy also improved the short-term treatment response in patients with other types of carcinoma apart from HCC (such as intrahepatic cholangiocarcinoma, locally advanced breast cancer and stage II–IV lung cancer) [19–21], especially in patients with gastrointestinal cancer liver metastasis [22, 23]. Inspired by this previous evidence, it was hypothesized that CalliSpheres® DEB-TACE therapy might also play a critical role in treating CRLM patients, especially in refractory cases failing standard chemotherapy or bevacizumab. However, no relevant study has yet been conducted.

Therefore, the current study aimed to compare the efficacy of CalliSpheres® DEB-TACE therapy versus cTACE therapy regarding the treatment response and survival profile in refractory CRLM (RCRLM) patients.

## Methods

### Participants

Between May 2018 and July 2020, 11 RCRLM patients who underwent DEB-TACE treatment and 11 RCRLM patients who underwent cTACE treatment in Yichang Central People's Hospital were analyzed in this retrospective study. The inclusion criteria were as follows: (1) pathologically confirmed colorectal cancer; (2) primary colorectal lesions were resected; (3) liver metastatic lesions were identified by imaging and laboratory tests; (4) liver metastatic lesions could not be treated by surgery or radiofrequency ablation; (5) refractory disease defined as having a history of systemic chemotherapy for primary colorectal lesions and could not receive chemotherapy and/or bevacizumab for metastatic lesions due to poor efficacy; and (6) Child‒Pugh stage A/B. The exclusion criteria were as follows: (1) complicated with metastasis at other sites apart from the liver; (2) > 70% of the liver was invaded with metastatic lesions; (3) Child‒Pugh stage C; and (4) Eastern Cooperative Oncology Group performance status (ECOG PS) score > 2. All participants or their family members signed written informed consent forms. This study was approved by the Ethics Committee of Yichang Central People's Hospital.

### Treatment procedures

The decision to receive DEB-TACE or cTACE was made based on the disease condition of the patients, the suggestion from the physicians, and the willingness of the patients. The current study did not intervene with the decision of treatment choice. The DEB-TACE and cTACE procedures were carried out in a digital subtraction angiography (DSA) room. First, the modified Seldinger technique was used to perform percutaneous right femoral artery puncture and intubation under the guidance of DSA. Then, a 5F Cobra (Cordis, Miami, USA) catheter was catheterized into the celiac trunk and superior mesenteric artery to determine the size, location distribution and blood supply of metastatic lesions in the liver. Then, a 2.7F microcatheter (Termbozus) was used to intubate the supplying artery of the lesions. After further confirming the supplying artery and location of the lesions by angiography, in the DEB-TACE group, CalliSpheres® Beads (300–500 μm, Jiangsu Hengrui Medicine Co. Ltd., China) loaded with 100 mg irinotecan were used to embolize the supplying artery; in the cTACE group, conventional materials mixed with 100 mg irinotecan were used to embolize the supplying artery. The embolization was stopped when the tumor staining completely disappeared.

### Evaluation of treatment response

Treatment response was evaluated by necrotic conditions of target metastatic liver lesions using enhanced computerized tomography (CT) or magnetic resonance imaging (MRI) based on modified Response Evaluation Criteria in Solid Tumors (mRECIST), which was categorized as CR, partial response (PR), stable disease (SD) and progressive disease (PD). The ORR was calculated as the sum of CR and PR; the DCR was defined as the sum of CR, PR and SD. All images were independently measured and evaluated by two associate chief radiologists; if a conflict appeared, a third chief radiologist was involved for judgment.

### Characteristics and follow-up

Characteristics of patients with RCRLM were collected from electronic medical records. Disease status and survival status were collected from follow-up data. Progression-free survival (PFS) was calculated from the date of initiation of DEB-TACE or cTACE treatment to the date of disease progression in the liver or death. Overall survival (OS) was calculated from the date of initiation of DEB-TACE or cTACE treatment to the date of death. The median follow-up duration was 14 (range 2–25) months in the DEB-TACE group and 7 (range 1–18) months in the cTACE group.

### Statistical analysis

Statistical analysis was performed using the SPSS 22.0 program (IBM, USA). Graphs were made using GraphPad Prism 7.01 software (GraphPad Inc., USA). The continuous data were expressed as the mean ± standard deviation (SD). Categorical variables are expressed as counts and percentages. Comparisons of characteristics between the DEB-TACE group and cTACE group were analyzed by independent-sample t test, Chi-square test, and Wilcoxon rank sum test. Comparison of the general clinical response between the DEB-TACE group and cTACE group was analyzed by the Wilcoxon rank sum test. Comparisons of ORR and DCR between the DEB-TACE group and cTACE group were analyzed by the chi-square test. Kaplan‒Meier curves were used to display PFS and OS. Differences in PFS and OS between the DEB-TACE group and cTACE group were determined by the log-rank test. Univariate and forward stepwise multivariate Cox proportional hazards regression models were used to assess factors affecting PFS and OS. Statistical significance was defined as *P* < 0.05.

## Results

### Study flow

**Fig. 1:**
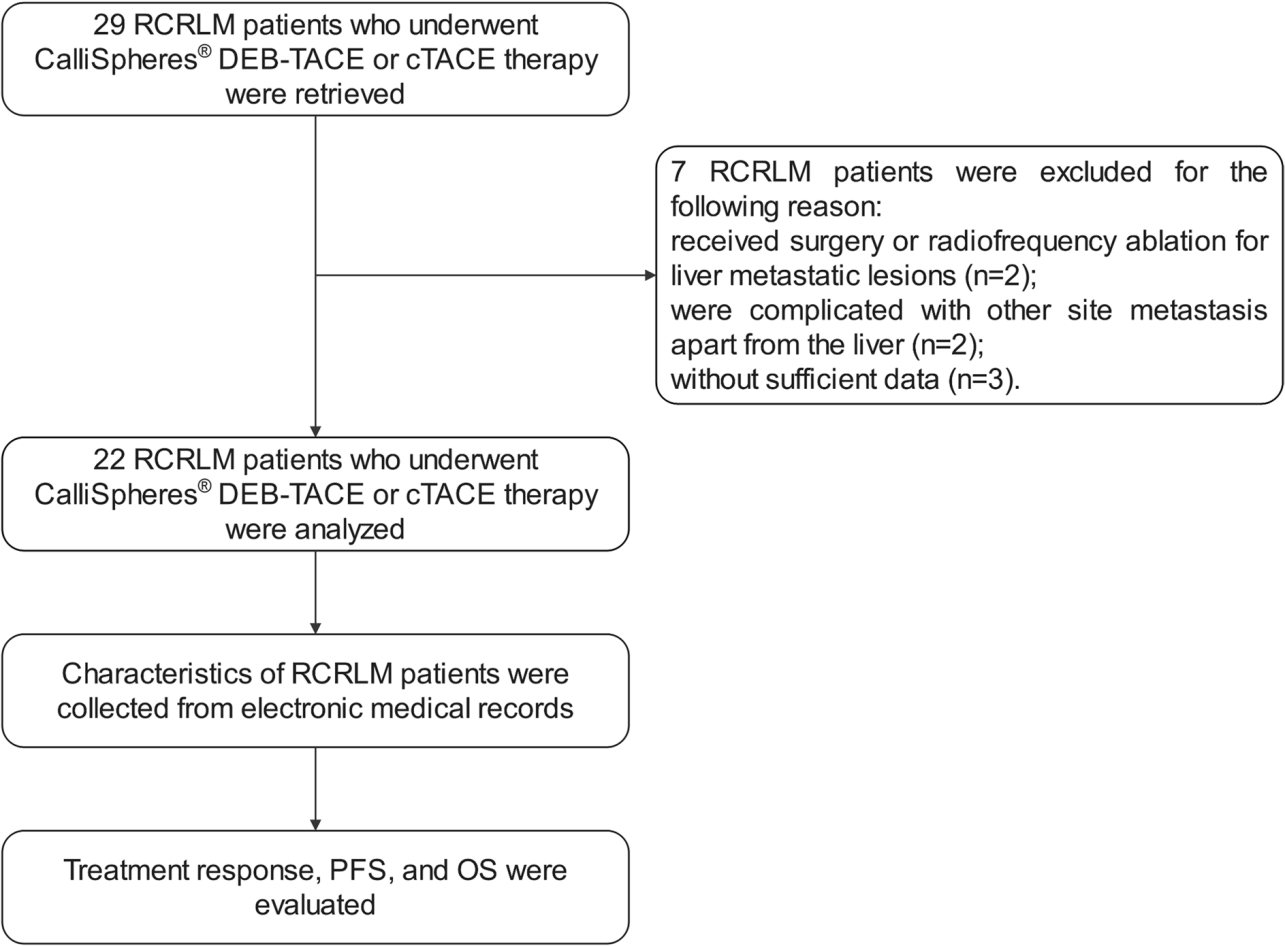
Consort diagram

In total, the information of 29 RCRLM patients who underwent CalliSpheres® DEB-TACE or cTACE was retrieved for evaluation of eligibility. Then, 7 patients were excluded from this study, including 2 patients who received surgery or radiofrequency ablation for liver metastatic lesions, 2 patients who were complicated with other sites of metastasis apart from the liver, 3 patients without sufficient data. Next, the remaining 22 patients were analyzed for treatment response, PFS, and OS (Fig. 1).

### Patient characteristics

The mean ages in the DEB-TACE group and cTACE group were 64.9 ± 8.4 years and 52.7 ± 14.1 years, respectively (Table 1). Compared to the cTACE group, the DEB-TACE group was older (*P* = 0.023). However, no differences in sex (*P* = 0.375), Child‒Pugh stage (*P* = 0.127), lesion distribution (*P* = 0.392), lesion size (*P* = 0.672), number of lesions *(P* = 0.647) or ECOG PS score (*P* = 1.000) were observed between the DEB-TACE group and the cTACE group. The detailed clinical features are shown in Table 1.

**Table 1:** Characteristics of patients with RCRLM

Parameters	DEB-TACE (*N* = 11)	cTACE (*N* = 11)	*P* value
Age (years), mean ± SD	64.9 ± 8.4	52.7 ± 14.1	0.023
Gender, No. (%)			0.375
Male	8 (72.7)	6 (54.5)	
Female	3 (27.3)	5 (45.5)	
Child‒Pugh stage, No. (%)			0.127
A	10 (90.9)	7 (63.6)	
B	1 (9.1)	4 (36.4)	
Lesion distribution, No. (%)			0.392
Single lobe	5 (45.5)	7 (63.6)	
Both lobes	6 (54.5)	4 (36.4)	
Lesion size, No. (%)			0.672
< 5 cm	6 (54.5)	7 (63.6)	
≥ 5 cm	5 (45.5)	4 (36.4)	
Number of lesions, No. (%)			0.647
Unifocal	3 (27.3)	4 (36.4)	
Multifocal	8 (72.7)	7 (63.6)	
ECOG PS score, No. (%)			1.000
0	0 (0.0)	2 (18.2)	
1	11 (100)	7 (63.6)	
2	0 (0.0)	2 (18.2)	

*RCRLM* Refractory colorectal cancer liver metastases; DEB-TACE, drug-eluting bead transarterial chemoembolization, *cTACE* Conventional transarterial chemoembolization, *SD* Standard deviation; *ECOG* Eastern Cooperative Oncology Group, *PS* Performance status

### Difference in treatment response between the DEB-TACE group and the cTACE group

**Fig. 2:**
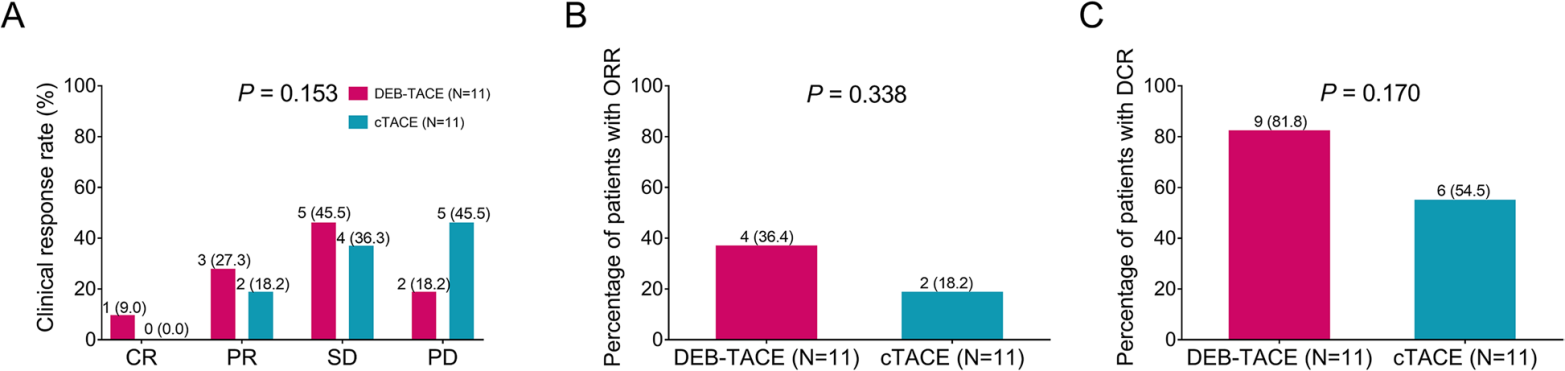
Comparison of treatment response between DEB-TACE and cTACE treatments in RCRLM patients. Comparison of total clinical response (**A**), ORR (**B**) and DCR (**C**) between DEB-TACE-treated patients and cTACE-treated patients

A total of 9.0%, 27.3%, 45.5%, and 18.2% of patients in the DEB-TACE group achieved CR, PR, SD and PD, respectively. In contrast, 0.0%, 18.2%, 36.3%, and 45.5% of patients in the DEB-TACE group achieved CR, PR, SD and PD, respectively (*P* = 0.153, Fig. 2A). Furthermore, the ORR was 36.4% in the DEB-TACE group and 18.2% in the cTACE group (*P* = 0.338, Fig. 2B). In addition, the DCR was 81.8% in the DEB-TACE group and 54.4% in the cTACE group (*P* = 0.170, Fig. 2C). Although no significant difference in total clinical response, ORR or DCR was observed between the DEB-TACE group and cTACE group, the DEB-TACE group displayed a higher trend of response rate partly due to the small sample size in our study.

### Differences in PFS and OS between the DEB-TACE group and the cTACE group

**Fig. 3:**
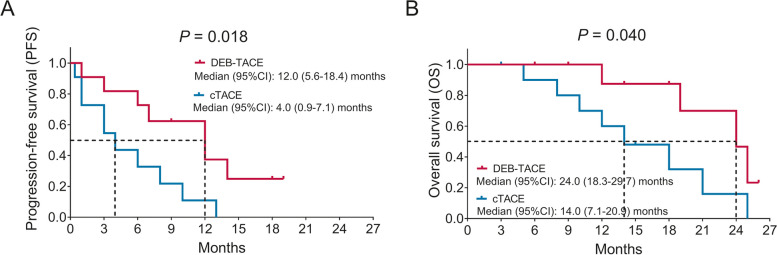
Comparison of survival profiles between DEB-TACE and cTACE treatments in RCRLM patients. Comparison of PFS (**A**) and OS (**B**) between DEB-TACE-treated patients and cTACE-treated patients

The median PFS was 12.0 months (95% CI: 5.6–18.4) in the DEB-TACE group and 4.0 months (95% confidence interval (CI): 0.9–7.1) in the cTACE group (Fig. 3A). In addition, the median OS was 24.0 months (95% CI: 18.3–29.7) in the DEB-TACE group but 14.0 months (95% CI: 7.1–20.9) in the cTACE group (Fig. 3B). Compared to the cTACE group, the DEB-TACE group achieved prolonged PFS (*P* = 0.018) and OS (*P* = 0.040).

### Cox regression model analysis of factors affecting PFS in RCRLM patients

Univariate Cox regression revealed that treatment option (DEB-TACE vs. cTACE) was correlated with better PFS in RCRLM patients (*P* = 0.027, hazard ratio (HR): 0.310, 95% CI: 0.110–0.875) (Table 2). Furthermore, multivariate Cox regression showed that treatment option (DEB-TACE vs. cTACE) was an independent predictive factor for prolonged PFS (*P* = 0.003, HR: 0.110, 95% CI: 0.026–0.463), while age (≥ 60 years vs. < 60 years) could independently predict worse PFS (*P* = 0.021, HR: 4.868, 95% CI: 1.264–18.751) in RCRLM patients.

**Table 2:** Cox proportional hazards regression model analysis of factors affecting PFS

Items	Cox’s proportional hazard regression model			
	*P* value	HR	95%CI	
			Lower	Higher
**Univariate Cox’s regression**				
Treatment (DEB-TACE vs. cTACE)	0.027	0.310	0.110	0.875
Age (≥ 60 years vs. < 60 years)	0.527	1.365	0.521	3.577
Gender (male vs. female)	0.939	1.039	0.392	2.754
Child‒Pugh stage (B vs. A)	0.506	1.494	0.458	4.873
Lesion distribution (both lobes vs. single lobe)	0.250	0.555	0.204	1.512
Lesion size (≥ 5 cm vs. < 5 cm)	0.398	0.655	0.245	1.748
Number of lesions (multifocal vs. unifocal)	0.134	0.445	0.154	1.283
ECOG PS score	0.436	1.699	0.448	6.447
**Forward stepwise multivariate Cox’s regression**				
Treatment (DEB-TACE vs. cTACE)	0.003	0.110	0.026	0.463
Age (≥ 60 years vs. < 60 years)	0.021	4.868	1.264	18.751

*PFS* Progression-free survival; *HR* Hazard ratio, *CI* Confidence interval, *DEB-TACE* Drug-eluting bead transarterial chemoembolization, *cTACE* Conventional transarterial chemoembolization, *ECOG* Eastern Cooperative Oncology Group, *PS* Performance status

### Cox regression model analysis of factors affecting OS in RCRLM patients

Univariate Cox regression revealed that treatment option (DEB-TACE vs. cTACE) was correlated with longer OS in RCRLM patients (*P* = 0.047, HR: 0.256, 95% CI: 0.067–0.982) (Table 3). In addition, multivariate Cox regression showed that treatment option (DEB-TACE vs. cTACE) was an independent predictive factor for improved OS (*P* = 0.006, HR: 0.126, 95% CI: 0.028–0.559), while sex (male vs. female) could independently predict shorter OS (*P* = 0.024, HR: 5.526, 95% CI: 1.250–24.435) in RCRLM patients.

**Table 3:** Cox proportional hazards regression model analysis of factors affecting OS

Items	Cox’s proportional hazard regression model			
	*P* value	HR	95%CI	
			Lower	Higher
**Univariate Cox’s regression**				
Treatment (DEB-TACE vs. cTACE)	0.047	0.256	0.067	0.982
Age (≥ 60 years vs. < 60 years)	0.847	1.119	0.356	3.522
Gender (male vs. female)	0.199	0.420	0.112	1.580
Child‒Pugh stage (B vs. A)	0.709	1.289	0.339	4.902
Lesion distribution (both lobes vs. single lobe)	0.416	0.591	0.166	2.103
Lesion size (≥ 5 cm vs. < 5 cm)	0.936	0.954	0.301	3.026
Number of lesions (multifocal vs. unifocal)	0.196	0.454	0.137	1.501
ECOG PS score	0.218	3.240	0.498	21.077
**Forward stepwise multivariate Cox’s regression**				
Treatment (DEB-TACE vs. cTACE)	0.006	0.126	0.028	0.559
Gender (male vs. female)	0.024	5.526	1.250	24.435

*OS* Overall survival, *HR* Hazard ratio, *CI* Confidence interval, *DEB-TACE* drug-eluting bead transarterial chemoembolization, *cTACE* Conventional transarterial chemoembolization, *ECOG* Eastern Cooperative Oncology Group, *PS* Performance status

## Discussion

DEB-TACE has been invented as a novel chemotherapy drug delivery system in treating patients with solid tumors, which displays several advantages: (i) the rounded sphere surface allows it to reach more distal arterioles, (ii) various microspheres with different diameters are able to embolize in different arteries with different luminal diameters, and (iii) these beads allow a controlled and sustained release of loaded anticancer chemotherapy drugs [24–26]. Apart from several advantages on its material and design, DEB-TACE therapy also exerts some functional improvements based on in vivo studies [27, 28]. For example, CalliSpheres® DEB-TACE therapy reduced systematic chemotherapeutic drug concentrations compared to cTACE therapy, indicating that it might cause less systematic cytotoxicity and a lower chance of adverse events [27]. Additionally, a previous in vivo study illustrated that CalliSpheres® DEB-TACE therapy reduced tumor volume and organ metastases (including pulmonary and bone) compared to cTACE therapy in liver cancer rabbits [28]. Taken together, CalliSpheres® DEB-TACE therapy displays certain improvement compared to cTACE therapy in treating liver cancer.

In terms of CRLM, DEB-TACE therapy using different microspheres also exerts an improved treatment response over cTACE or chemotherapy. For instance, DEB-TACE treatment using degradable starch microspheres displayed a higher response rate and lower tumor volume than cTACE treatment in CRLM patients [29]. In addition, DEB-TACE therapy using DC Beads® showed an improved ORR at each time point (M2, M4 and M6) compared to systemic chemotherapy in CRLM patients [30]. However, the efficacy of CalliSpheres® DEB-TACE for CRLM treatment is seldom reported. Therefore, we conducted this study and discovered that CalliSpheres® DEB-TACE therapy displayed a higher trend of clinical response than cTACE (although not reaching statistical significance) in RCRLM patients. The possible reasons for this were as follows: (a) The sample size in our study was relatively small, which might reduce statistical power, so further study with a larger sample size is necessary to validate this. (b) Patients who received CalliSpheres® DEB-TACE therapy were older than those who received cTACE treatment, thereby leading to a higher risk of atherosclerosis causing occlusion of distal vessels, which might decrease the chemotherapy concentration in those distal arterioles and result in insignificant treatment response rates between the two treatments. (c) The recruited RCRLM patients in our study received systemic chemotherapy and colectomy surgery prior to TACE treatment, which might induce bias and lead to an insignificant treatment response between the two treatments.

Additionally, the survival profile of DEB-TACE therapy using different microspheres in CRLM patients is of great interest as well. For example, DC beads® DEB-TACE therapy displayed a prolonged PFS compared to systematic chemotherapy in CRLM patients; however, there was no difference in OS between DEB-TACE and cTACE operations [31]. Meanwhile, another interesting study reported that degradable starch microsphere DEB-TACE therapy showed a trend of longer OS than cTACE therapy in CRLM patients [29]. Partially in line with these studies, our study observed an improvement in the survival profile with CalliSpheres® DEB-TACE therapy compared to cTACE therapy in RCRLM patients. Additionally, treatment option (CalliSpheres® DEB-TACE therapy vs. cTACE therapy) independently correlated with prolonged PFS and OS in RCRLM patients. The possible reasons could be explained as follows: (a) Compared to cTACE therapy, CalliSpheres® DEB-TACE therapy might induce a sustained release of loaded chemotherapeutic drug as well as lower systematic cytotoxicity, thereby leading to a favorable survival profile in RCRLM patients [16, 17]. (b) CalliSpheres® DEB-TACE therapy might suppress tumor angiogenesis owing to its local higher chemotherapeutic drug concentration in the target tumor site, thereby further leading to a prolonged survival profile over cTACE therapy in RCRLM patients [28]. (c) Compared to cTACE therapy, CalliSpheres® DEB therapy had various microspheres with different diameters, which were able to embolize different sizes of arteries more suitably, thereby leading to prolonged OS and PFS in RCRLM patients [32]. Taken together, CalliSpheres® DEB-TACE therapy improved the survival profile of RCRLM patients and might act as a promising treatment option.

In the current study, we excluded 2 patients who received surgery or radiofrequency ablation for liver metastatic lesion since these were radical treatment options for liver metastatic lesion, and the survival benefit in these patients was mainly due to surgery or radiofrequency ablation, rather than DEB-TACE or cTACE. Meanwhile, another 2 patients with other site metastasis apart from liver (mainly lung) since the survival of these patients mainly depended on the lung metastatic site. The exclusion of these patients might better focus on the treatment response of DEB-TACE or cTACE; however, this would induce potential bias. There were some other limitations in the present study. First, the sample size in our study was relatively small; thus, further study with a larger sample size is needed. Second, the follow-up period in our study was relatively short; thus, the long-term efficacy of CalliSpheres® DEB-TACE therapy could not be determined in our study, and further study is necessary. Third, as a retrospective cohort study, our study did not directly randomize patients’ treatment, and some confounding factors existed that interfered with the results; thus, further randomized controlled trials are needed to validate the findings. Fourth, this was a single-center study, which would inevitably induce regional bias. Therefore, further multicentric studies should be performed for verification.

In conclusion, CalliSpheres® DEB-TACE therapy may improve the survival profile compared to cTACE therapy to some extent in RCRLM patients, suggesting its potency as a treatment option for RCRLM management. However, further multicentric, large-scale, randomized, controlled trials should be performed to verify the efficacy of CalliSpheres® DEB-TACE in patients with RCRLM.

## Data Availability

The datasets used and/or analyzed during the current study are available from the corresponding author on reasonable request.
